# Optic Disc Hemorrhage Is Not Associated with Global Choroidal Vessel Loss, but Is Associated with Localized Choroidal Vessel Loss in Glaucoma

**DOI:** 10.3390/jcm11041080

**Published:** 2022-02-18

**Authors:** Anna Lee, Joong Won Shin, Jin Yeoung Lee, Min Su Baek, Michael S. Kook

**Affiliations:** Department of Ophthalmology, Asan Medical Center, College of Medicine, University of Ulsan, 88, Olympic-Ro 43-Gil, Songpa-Gu, Seoul 05505, Korea; hooni811@naver.com (A.L.); sideral@hanmail.net (J.W.S.); gnyoungee@naver.com (J.Y.L.); bmasiuenk@daum.net (M.S.B.)

**Keywords:** glaucoma, optical coherence tomography angiography, optic disc hemorrhage, parapapillary choroidal vessel density

## Abstract

Purpose: To investigate the relationship between optic disc hemorrhage (ODH) and the geographic pattern (regional vs. global) of parapapillary choroidal vessel density (pCVD) loss within the β-parapapillary atrophy (β-PPA) in open-angle glaucoma (OAG) Methods: This retrospective cross-sectional study included 100 OAG eyes with visual field (VF) defects confined to a single hemifield (50 with and 50 without ODH, matched for age (≤10 years) and VF severity (≤1 decibel) at the same hemifield), as well as 50 healthy eyes. The pCVD was measured using optical coherence tomography angiography (OCTA). The relationships between pCVD and clinical factors were assessed globally and regionally. Logistic regression analyses were performed to determine the clinical factors associated with the presence of ODH. Results: The pCVD values within ODH-affected hemiretinae of ODH+ eyes were significantly lower than those in the matched hemiretinae of ODH− eyes (*p* = 0.001). The presence of ODH was significantly correlated with a lower pCVD within ODH-dominant inferior hemiretinae (*p* < 0.05). Lower pCVD values at ODH-affected hemiretinae was significantly associated with the presence of ODH (*p* < 0.05). Conclusions: OAG eyes with ODH demonstrate a greater severity of regional pCVD loss at the hemiretinae spatially corresponding to the ODH location compared to OAG eyes without ODH.

## 1. Introduction

The recent introduction of optical coherence tomography angiography (OCTA) has allowed clinicians to noninvasively image the microvasculature of the optic nerve head (ONH), retina, and choroid and evaluate the perfusion status of these structures. Several studies using OCTA have revealed diminished ONH/retinal vessel density (VD) in eyes with glaucoma [1,2,3,4,5]. Moreover, microvasculature dropout in the parapapillary choroid (CMvD), defined as a complete loss of the choriocapillaries and choroidal microvasculature network within the β-parapapillary atrophy (β-PPA), is associated with both retinal nerve fiber layer (RNFL) defects and visual field (VF) damage with spatial correlations and progressive glaucomatous damage [2,3,6]. Because the parapapillary choroid has an extensive interconnection of microvasculature networks within the β-PPA [7,8,9], CMvD may indicate global or diffuse loss of perfusion to the parapapillary choroid in glaucomatous eyes [10].

Optic disc hemorrhage (ODH) has been considered a well-known risk factor for progressive VF loss and RNFL thinning in glaucoma [11,12,13,14,15]. In addition, ODH correlates topographically with structural defects such as β-PPA, a larger cup–disc ratio (CDR), lamina cribrosa (LC) defects, and a worsening of the rate of RNFL thinning in a ODH-associated quadrant [14,16,17]. Although the pathogenesis of ODH is not yet fully understood, mechanical vascular disruption at the level of the LC or the margin of RNFL defect may be related to the occurrence of ODH [17,18]. In contrast, vascular insufficiency such as altered ocular perfusion pressure resulting from primary vascular dysregulation, decreased ocular blood flow, and/or unstable systemic blood pressure (BP) may be also linked to the detection of ODH [19,20].

The prevalence of ODH is significantly higher in eyes with CMvD than in eyes without CMvD [21,22]. Moreover, there is a topographic correlation between ODH and the CMvD location in the glaucomatous ONH [21,22]. These findings suggest that both ODH and CMvD may share the vascular manifestations of glaucomatous damage. We hypothesized that ODH would be associated with perfusion reduction in the parapapillary choroid similar to CMvD. While the presence of CMvD is associated with a global or diffuse reduction in parapapillary choroidal VD (pCVD) within the β-PPA zone [10], the pattern of choroidal VD loss (i.e., regional vs. global) in open-angle glaucoma (OAG) eyes with ODH has not been fully investigated to date. Determining this pattern may provide an insight into the risk profiling of ODH in glaucoma progression, as more generalized vascular insufficiency in the parapapillary choroid may indicate greater vulnerability to future glaucomatous progression. The aim of our present study was to investigate whether there is a geographic predilection (i.e., regional vs. global) for pCVD loss associated with ODH by examining the differences in the global and regional pCVD values between the OAG eyes with ODH and those without ODH, matched for age and severity of VF damage using OCTA measures.

## 2. Materials and Methods

### 2.1. Study Subjects

This retrospective study was approved by the Institutional Review Board (IRB) of the Asan Medical Center, which waived the requirement for informed consent from participants. All procedures conformed to the tenets of the Declaration of Helsinki.

We conducted a consecutive review of the medical records of patients who visited our glaucoma clinic at Asan Medical Center from November 2019 to April 2021. During the initial glaucoma work-up, all subjects underwent comprehensive ophthalmic examinations, including a review of their medical history, a measurement of best-corrected visual acuity (BCVA), slit-lamp biomicroscopy, Goldmann applanation tonometry, refractive error, axial length (AL) measurement (IOL Master version 5; Carl Zeiss Meditec, Dublin, CA, USA), ultrasound pachymetry (DGH-550; DGH Technology, Inc., Exton, PA, USA) for central corneal thickness (CCT) measurement, dilated color fundus photography (Canon, Tokyo, Japan), optic disc stereo-photography and red-free RNFL photography (Canon). These patients also underwent Humphrey field analyzer (HFA) Swedish Interactive Threshold Algorithm (SITA)-Standard 24-2 VF testing (Carl Zeiss Meditec), measurement of circumpapillary RNFL thickness (cpRNFLT) using spectral-domain optical coherence tomography (SD-OCT; Cirrus HD; Carl Zeiss Meditec), and imaging with a commercial OCTA system (Angiovue; Optovue Inc., Fremont, CA, USA). Systolic and diastolic BP were also measured during the initial evaluation. The mean ocular perfusion pressure (MOPP) was estimated as the difference between two-thirds of the mean arterial pressure (MAP) and intraocular pressure (IOP). The MAP was calculated as the summation of one-third of the systolic and two-thirds of the diastolic BP.

Participants enrolled in our study included those with OAG as well as normal healthy subjects. Both the OAG and healthy subjects included in our analyses needed to satisfy the following criteria: (1) age ≥ 18 years; (2) BCVA ≥ 20/30; (3) spherical equivalent (SE) of between −6.0 and +3.0 diopters (D) and a cylinder correction within ±3D; (4) reliable VF testing (false-positive errors <15%, false-negative errors <15%, and fixation loss <20%); (5) normal anterior and posterior chamber; and (6) open angles on slit lamp exam and gonioscopy. In addition, the OAG subjects were required to meet the following conditions for inclusion in the study cohort: (1) the presence of a glaucomatous optic disc change (i.e., focal or generalized neural rim loss and localized or diffuse atrophy of the RNFL) with a visible β-PPA on fundus photography and compatible glaucomatous VF defects, irrespective of the IOP level; (2) glaucomatous VF defects defined by Anderson’s criteria [13]. To account for the learning effects in VF testing, a second VF test was obtained within one week if the first VF result was judged to be glaucomatous; and (3) early-stage glaucomatous VF defects confined to a single hemifield (superior or inferior) with mean deviation (MD) better than −6 decibels (dB). Early-stage OAG eyes were selected in the present study, as ODH is most frequently found in early-stage OAG eyes with VF defects confined to a single hemifield [23,24] and pCVD measurement using OCTA is affected by glaucoma severity in which the more advanced the glaucoma, the greater and more diffuse the choroidal VD loss [2,10,22]. The normal healthy participants consisted of subjects from the general eye clinic matched to OAG eyes by age (≤10 years) in order to minimize the confounding effect of age on VD measurements [10,22] and were required to have (1) an IOP < 21 mmHg with no history of an elevated IOP; (2) a normal-appearing ONH with a visible β-PPA on fundus photography; (3) a normal cpRNFLT (average and quadrant cpRNFLT within 99% confidence limits) based on SD-OCT; and (4) normal VF test results (i.e., a PSD within 95% confidence limits and a GHT result within normal limits) [13].

Subjects were excluded if they had one or more of the following conditions: high myopia with an SE < −6 D and/or AL > 26 mm, severe myopic disc and fundus changes and/or posterior staphyloma that may impair adequate ONH/VF/SD-OCT/OCTA evaluation; a media opacity including cataracts of more than C2, N2, or P2 based on the Lens Opacities Classification System III [25]; a history of intraocular surgery (including any cataract/glaucoma operations), a history of trauma, a history of systemic or neurologic conditions, or any other ophthalmic diseases that could affect ONH/VF evaluation, including retinal detachment, diabetic retinopathy, or retinal vascular occlusive diseases. If both eyes were eligible in any subject, one eye was randomly selected for the study.

### 2.2. Pairing of the ODH+ and ODH− OAG Groups and Hemiretinal ODH Locations

**Figure 1:**
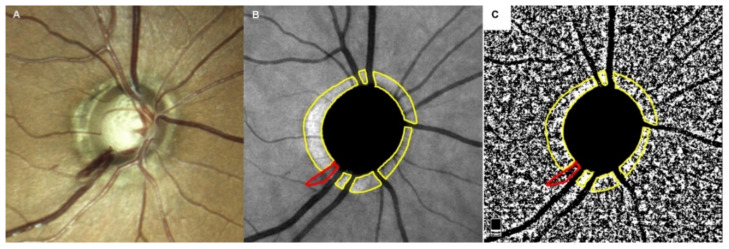
Measurement of the parapapillary choroidal vessel density (pCVD). (**A**) The right eye of an open-angle glaucoma subject is shown with a splint-shape optic disc hemorrhage (ODH) at the inferior temporal side of the optic disc (7 o’clock) on a stereoscopic optic disc photograph, which appeared to cross the disc margin on the border of the retinal nerve fiber layer defect. (**B**) Optic disc and β-parapapillary atrophy margins, excluding large retinal vessels and ODH, were manually demarcated (yellow outline) using ImageJ software on a scanning laser ophthalmoscopy image obtained by optical coherence tomography angiography. (**C**) The ODH area at the same location of the scanning laser ophthalmoscopy image (red outline) along with large retinal vessels were excluded in eyes with ODH for the measurement of pCVD. The selected areas of interest after removal of the ODH area and large retinal vessels were converted to range of interest and applied to the 8-bit binary slab of en face choroidal layer image to derive the pCVD measurement.

Glaucomatous ODH was defined in this present study as an isolated flamed- or splinter-shaped hemorrhage located in the peripapillary area or on the optic disc (Figure 1A) [26]. OAG eyes exhibiting a single ODH and VF defects confined to a single hemifield (superior or inferior) were selected from the cohort of OAG eyes that met the initial inclusion criteria. ODH was confirmed based on the presence of ODH at the time of OCTA evaluation or anytime in the past by reviewing the past medical records including optic disc stereo-photographs when available. From the same OAG database, OAG eyes with no documented ODH history (ODH− eyes) were matched to OAG eyes with ODH (ODH+ eyes) with respect to the age (≤10 years) and VF loss (≤1 dB) at the same hemifields.

To define the hemiretinal location of ODH in the present study series, a reference line was drawn from the optic disc center to the center of the macula on a color fundus photograph. The center of the macula was defined as a punctate central reflex of the band-like reflex on a color fundus photograph. This reference line was used to divide the hemiretinae and assign each ODH to a superior or inferior location [10]. Eyes with ODHs that were located on the reference line were excluded from the analysis. The presence and location of an ODH in each case was independently assessed by two glaucoma specialists (A.L. and J.Y.L.) during the initial classification process, who were blind to the clinical data for the patients. Any disagreements between the two observers in terms of presence and location of ODH were resolved by a third adjudicator (M.S.K.).

### 2.3. OCTA and Parapapillary Choroidal Vessel Density Measurements

OCTA imaging of the ONH was performed using the AngioVue OCTA system (Optovue Inc.). This OCTA system utilizes the split-spectrum amplitude-decorrelation angiography method to capture the dynamic motion of the red blood cells and provide high-resolution 3-dimensional visualization of microvascular structures [27]. All OCTA parameters from this study were extracted from the same version of AngioVue software (version 2018.1.0.43) to ensure data consistency. The choroidal microvasculature in the parapapillary area was evaluated on en face images generated by layer segmentation of signals from the retinal pigment epithelium to 390 µm below Bruch’s membrane [3,10]. Briefly, boundaries of the optic disc and β-PPA were first manually delineated using ImageJ software [3,10], while excluding large projecting retinal vessels and ODH area within the β-PPA zone on scanning laser ophthalmoscopy images of ODH+ eyes (Figure 1B) [10]. The optic disc and β-PPA margins were then overlaid after matching the vessel blanching on an en face choroidal layer image of the OCTA. After the selected area of interest on the en face choroidal layer image was binarized using the mean binarization method, the vascularization area was described as the white pixel [3,10]. pCVD was defined as the percentage of white pixels relative to the total number of pixels within the β-PPA zone (Figure 1C).

Hemiretinal readings of the pCVD as well as global pCVD measurement within the β-PPA zone were obtained according to the method described by Jo et al. [10]. Since there is currently no gold standard for the measurement of regional vs. global pCVD within the β-PPA zone, hemiretinal pCVD readings were used to represent the regional pCVD in relation to ODH. For paired OAG eyes (i.e., an ODH+ vs. ODH− eye) and matched healthy control eyes, two hemiretinal measurements of the pCVD were analyzed at the superior and inferior hemiretinae within the β-PPA. In the matched three groups (ODH+, ODH−, and healthy control eyes), the pCVDs that measured in the same hemiretinae as the ODH location in the ODH+ eyes were defined as ODH-affected hemiretinal pCVDs. Conversely, pCVDs measured in the hemiretinae without ODH in the ODH+ eyes were defined as ODH-unaffected hemiretinal pCVDs.

The presence of CMvD was assessed using en face choroidal OCTA imagery. The CMvD was defined as a focal and complete loss of both the choriocapillaris and choroidal microvasculature with no visible microvasculature network within the β-PPA, with a minimum angular width of 200 μm or greater at any location [6,10,22]. In the eyes with CMvD and overlying ODH, the presence of CMvD was confirmed only when the CMvD was visible, and its size was larger than that of ODH on en face choroidal OCTA image. Any disagreements between the two observers were resolved by a third adjudicator (M.S.K.). All en face choroidal layer images were evaluated independently by the two glaucoma specialists (A.L. and J.Y.L.), who identified the margins of the optic disc, β-PPA, ODH, and CMvD, while blind to the clinical, VF, and SD-OCT information of the study subjects. The derived pCVD data from the two glaucoma specialists were averaged and used in the final analysis to minimize inter-observer variability.

### 2.4. Circumpapillary Vessel Density Measurements

**Figure 2:**
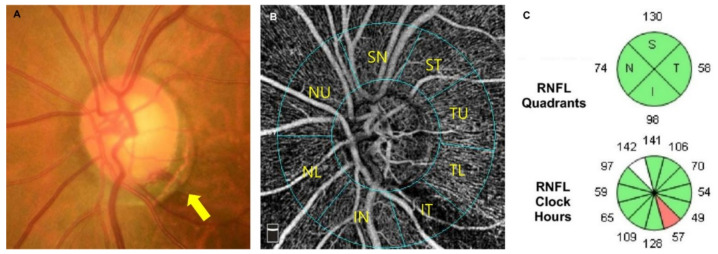
Measurement of the circumpapillary vessel density (cpVD) and circumpapillary retinal nerve fiber layer thickness (cpRNFLT). (**A**) Left eye of an open-angle glaucoma subject showing a splint-shape optic disc hemorrhage (ODH) at the inferior temporal side of optic disc (7 o’clock) on an optic disc photograph (yellow arrow). (**B**) A 4.5 mm × 4.5 mm en face optical coherence tomography (OCT) angiography image centered on the optic nerve head. The peripapillary area was divided into eight sectors in accordance with the Garway-Heath map [28]. The cpVD measured in the same hemiretinae as the ODH location (inferior hemiretinae) was defined as the ODH-affected hemiretinal cpVD, while the cpVD measured in the hemiretinae without ODH (superior hemiretinae) was defined as the ODH-unaffected hemiretinal pCVD. For the eight 45° sectoral measurements, the cpVD measured at the inferior temporal sector (IT) where the ODH was detected was defined as an ODH-affected sectoral cpVD, while that measured at the sector located symmetrical to the horizontal line (superior temporal sector: ST) was defined as an ODH-unaffected sectoral cpVD. (**C**) cpRNFLT quadrant and clock-hour map of spectral domain OCT. The cpRNFLT measured at the inferior quadrant was defined as the ODH-affected hemiretinal cpRNFLT, while that measured at the superior quadrant was defined as the ODH-unaffected hemiretinal cpRNFLT (upper section). The cpRNFLT measured at 7 o’clock on a clock-hour map was defined as the ODH-affected sectoral cpRNFLT, while that measured at the sector located symmetrical to the horizontal line (11 o’clock) was defined as the ODH-unaffected sectoral cpRNFLT (lower section). IN, inferior nasal; IT, inferior temporal; NL, nasal lower; NU, nasal upper; SN, superior nasal; ST, superior temporal; TL, temporal lower; TU, temporal upper.

Circumpapillary VD (cpVD) measurements were made using images of 4.5 × 4.5 mm^2^ scans centered on the optic disc within the radial peripapillary capillary slab from the internal limiting membrane to the nerve fiber layer after the automated removal of large retinal vessels (Figure 2A,B). The cpVD was defined as the percentage of the measured area occupied by small vessels within an instrument-defined 1000 µm-wide elliptical annulus from the optic disc boundary. The software automatically provides regional cpVD of the superior and inferior hemiretinae. In addition, the peripapillary region was divided into eight 45° sectors, i.e., temporal upper (TU), superior temporal (ST), superior nasal (SN), nasal upper (NU), nasal lower (NL), inferior nasal (IN), inferior temporal (IT), and temporal lower (TL), in accordance with the modified Garway-Heath map (Figure 2B) [28]. For the eight 45° sectoral measurements, the cpVD in the sector where the ODH was detected in the ODH+ eye was defined as ODH-affected sectoral cpVD, while the cpVD measured in the sector located symmetrically to the horizontal line was defined as ODH-unaffected sectoral cpVD (i.e., IT vs. ST sector) in each eye of each ODH+/ODH− pair and matched healthy control eye. OCTA images were excluded from the analysis if they had any of the following quality issues: (1) signal strength index <7; (2) poor clarity due to media opacity; (3) motion artifacts visualized as an irregular vessel pattern or disc boundary on an en face image; (4) localized weak signal intensities due to floater or posterior vitreous detachment; (5) images with a fixation error; or (6) segmentation failure [29].

### 2.5. SD-OCT Assessments

The cpRNFLT was measured using Cirrus HD SD-OCT software, version 10.0 (Carl Zeiss Meditec). This software automatically provides the cpRNFLT values globally, for each sector of four quadrants, as well as clock-hour assessment (Figure 2C). Superior (S) and inferior (I) quadrant measurements of the cpRNFLT were used to represent the cpRNFLT of two hemiretinal region (i.e., ODH-affected and -unaffected hemiretinae). In each ODH+ and ODH− pair and matched healthy control eye, the cpRNFLTs measured in the same quadrant as the ODH location in the ODH+ eyes were defined as ODH-affected quadrant cpRNFLTs, while those measured at the quadrant opposite to the ODH-affected quadrant (i.e., I vs. S quadrant) was defined as ODH-unaffected quadrant cpRNFLTs. cpRNFLTs measured at the clock hour where the ODH was located was defined as ODH-affected clock-hour cpRNFLTs, while the cpRNFLTs measured at the clock hour located symmetrical to the horizontal line were defined as the ODH-unaffected clock-hour cpRNFLTs (i.e., 7 o’clock vs. 11 o’clock). Only SD-OCT scans with good centration and high signal strength (SS) ≥7 and with adequate clarity and no localized weak signals caused by artifacts such as floaters, motion artifacts, and/or segmentation errors, were included.

### 2.6. Statistical Analysis

All statistical analyses were performed using SPSS, version 21.0 (IBM Corp, Chicago, IL, USA) and *p* values less than 0.05 were considered statistically significant. Results are presented as mean values with a standard deviation (SD) or as a frequency and percentage. The inter-observer agreement (A.L., J.Y.L.) for the presence and location of an ODH, presence of CMvD, and global pCVD value was assessed using the kappa (k) statistic and the intraclass correlation coefficient (ICC), respectively. The normality of distribution was assessed using the Kolmogorov–Smirnov test. The demographic and clinical characteristics of the study subjects were compared among the ODH+, ODH−, and healthy control eye groups. Comparisons among groups were performed using one-way analysis of variance with the Tukey’s post hoc test for quantitative variables and chi-square test with the Bonferroni correction for categorical variables.

Clinical variables associated with the pCVD values of the global area and the inferior and superior hemiretinal region within the β-PPA zone in OAG eyes were analyzed using univariate and multivariate linear regression analysis. Variables with *p* values < 0.05 in the univariate analysis were included in the multivariate linear regression analyses with a backward elimination process. The cpVD and cpRNFLT values were separately analyzed in the multivariate analysis to avoid the collinearity effect.

Both univariate and multivariate logistic regression analysis were used to determine the association between ODH as a dependent variable and various clinical factors in OAG eyes. Among independent variables, both global and regional parameters of ODH-affected and ODH-unaffected pCVD/cpVD/cpRNFLT were entered. A backward elimination process was used to build the multivariate analysis with variables found to be significant in the univariate analyses (*p* < 0.05). To avoid the effect of multi-collinearity among the pCVD, cpVD and cpRNFLT results, three different multivariate models were separately constructed.

## 3. Results

After an initial review of our database, a total of 190 eyes, made up of 52 healthy eyes and 138 OAG eyes (68 ODH+ eyes and 70 ODH− eyes), were selected for our current analyses. Of these initial cases, three eyes with unreliable VF testing and 37 eyes with poor quality OCT/OCTA scans were excluded. Hence, our final cohort included 150 eyes consisting of 50 healthy eyes and 50 ODH+ and 50 ODH− eyes matched by the VF loss at the same hemifields and age. There were excellent inter-observer agreements for the determination of the presence and location of an ODH and the presence of CMvD (k = 0.957, 0.911, and 0.90, respectively). The inter-observer ICC for the measurements of the global pCVD was 0.904 (95% CI: 0.871 to 0.938).

Comparisons of the demographics and baseline clinical characteristics among the ODH+, ODH− and healthy eyes are summarized in Table 1. In this study, the follow-up time was defined as the period from the day when a ODH was detected to the day of OCTA evaluation in ODH+ eyes and as the period from the first day of visit to the day of OCTA evaluation in ODH− eyes. The follow-up periods for the review of past medical history of eyes with and without ODH were 0.51 and 4.19 years, respectively. In the ODH+ group, inferior hemiretinae were ODH-dominant hemiretinae, as 36 eyes (72%) in the inferior hemiretinae and 14 eyes (28%) in the superior hemiretinae showed ODHs, respectively. The prevalence of CMvD was observed in 19 eyes (38%) in the ODH+ group and in nine eyes (18%) in the ODH− group, which was statistically significant (*p* < 0.001).

Comparisons of the OCT/OCTA-derived parameters are presented in Table 2. Compared with the healthy group, the eyes in both the ODH+ and ODH− groups showed a significantly lower global cpRNFLT, cpVD and pCVD (all *p* < 0.001). However, there were no significant differences in global cpRNFL, cpVD or pCVD between the ODH+ and ODH− eyes (*p* > 0.05). Notably, in the comparison of regional OCT/OCTA-measured parameters, the pCVD values for the ODH-affected hemiretinae in ODH + eyes were significantly lower than those for the ODH− group (*p* = 0.01), whereas there was no significant difference found between the pCVD values at ODH-unaffected hemiretinae in the two groups (*p* > 0.05). In addition, ODH+ eyes showed a significantly lower cpRNFLT for the ODH-affected clock hour compared to ODH− eyes (*p* < 0.001). The cpVD values for the ODH-affected sector and for the ODH-affected hemiretinae were significantly lower in the ODH+ eyes compared with the ODH− eyes (both *p* < 0.05). In contrast, there were no significant differences evident in the cpRNFLT and cpVD values for ODH-unaffected sectors between the ODH+ eyes and ODH− eyes (*p* > 0.05).

Table 3 presents the univariate and multivariate linear regression results for clinical factors associated with global pCVD in the entire 100 OAG eyes. In the univariate linear regression analysis, the presence of CMvD, AL, global cpRNFLT and global cpVD were significantly associated with global pCVD (*p* < 0.05). In the two separate multivariate models, the presence of CMvD and global cpVD were significantly associated with global pCVD in model 1 (*p* < 0.001 and *p* = 0.048, respectively), while global cpRNFLT and AL were significantly associated with global pCVD in model 2 (*p* < 0.001 and *p* = 0.044, respectively).

Table 4 indicates the univariate and multivariate linear regression results for determining the association between clinical factors and regional pCVD as dependent variables at inferior and superior hemiretinae in the entire OAG eyes. While the presence of ODH and CMvD, and AL were found to be significantly associated with pCVD at ODH-dominant inferior hemiretinae in both multivariate models 1 (Model 1: *p* = 0.011, *p* < 0.001, and *p* = 0.025, respectively; Table 4, upper section), the presence of CMvD and global cpVD were significantly associated with pCVD at superior hemiretinae (ODH-deficient hemiretinae) in multivariate model 1 (*p* = 0.003 and *p* = 0.005, respectively; Table 4, lower section).

As indicated in Table 5, a lower pCVD at ODH-affected hemiretinae (Model 1: *p* = 0.001), lower cpVD at an ODH-affected sector (Model 2: *p* = 0.024), and lower cpRNFLT at an ODH-affected clock hour (Model 3: *p* = 0.009), were significantly associated with the presence of ODH in multivariate analysis. None of the global OCT/OCTA parameters were associated with the presence of ODH.

**Figure 3:**
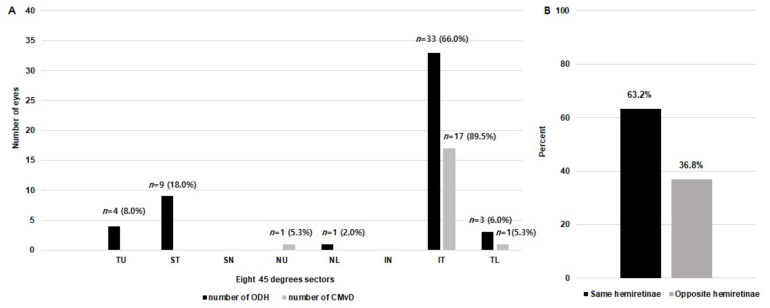
(**A**) Bar chart illustrating the number of eyes with optic disc hemorrhage (ODH: black bars) and choroidal microvasculature dropout (CMvD: gray bars). Data were converted to right-eye format. (**B**) Percentage of eyes with spatial concordance between ODH and CMvD detection. ODH, optic disc hemorrhage; CMvD, choroidal microvasculature dropout; IN, inferior nasal; IT, inferior temporal; NL, nasal lower; NU, nasal upper; SN, superior nasal; ST, superior temporal; TL, temporal lower; TU, temporal upper.

As shown in Figure 3, CMvDs were detected primarily at the IT (17 eyes, 89.5%), followed by TL (1 eyes, 5.3%), and NU (1 eyes, 5.3%) locations of the optic disc, according to the modified Garway-Heath map. There were 35 ODHs observed primarily in the IT (33 eyes, 66%), followed by ST (nine eyes, 18%), TU (four eyes, 8%), TL (three eyes, 6%) and NL (one eye, 2%) location of the optic disc (Figure 3A). Of the 19 CMvDs in the ODH+ eyes, 12 CMvDs (63.2%) were located in the same hemiretinae as the ODH, while seven CMvDs (36.8%) were observed in the opposite hemiretinae as the ODH (Figure 3B).

**Figure 4:**
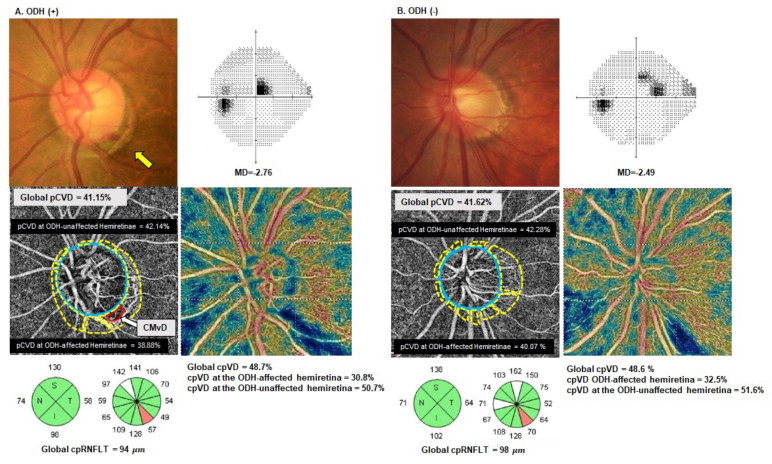
(**A**) representative matched case pairing with optic disc hemorrhage (ODH; (**A**)) and without ODH (**B**). Yellow and white arrows in (**A**) indicate ODH and choroidal microvasculature dropout (CMvD) indicated by a red outline, respectively. The blue outlines indicate the optic disc margin. In each group, the parapapillary choroidal vessel density (pCVD) was measured within the β-parapapillary atrophy which is demarcated by yellow outlines. The representative case shown in (**A**) was a 65-year-old male with a visual field (VF) defect confined to the superior hemifield in his left eye (VF mean deviation (MD) = −2.76 dB). ODH was detected at the inferior temporal (IT) location (5 o’clock) on stereoscopic optic disc photographs (yellow arrow). The en face choroidal image of the optical coherence tomography angiography (OCTA) scan showed a CMvD at the inferior temporal location where the ODH was located (white arrow). The representative case shown in (**B**) was a 58-year-old male without ODH, matched to the case in (**A**) by glaucoma severity (VF MD = −2.49 dB), location of the VF defect (i.e., confined to the superior hemifield) and age (≤10 years). Compared with the ODH− eye, the ODH+ eye in this pairing showed a lower pCVD at the ODH-affected hemiretinae (inferior), lower circumpapillary vessel density (cpVD) at the ODH-affected sector (IT), and thinner circumpapillary retinal nerve fiber layer thickness (cpRNFLT) at the ODH affected clock hour (7 o’clock). In OCTA measurements, the pCVD at the ODH-affected hemiretinae was 38.88% vs. 40.07%, the cpVD at the IT was 30.8% vs. 32.5%, and the cpRNFLT at ODH-affected clock hour was 57 μm vs. 70 μm, respectively, for the ODH+ eye compared with the ODH− eye. In contrast, the global pCVD, cpVD, and cpRNFLT in the ODH+ eye were similar to those in ODH− eyes, i.e., 41.15% vs. 41.62%, 48.7% vs. 48.6%, and 94 μm vs. 98 μm, respectively. Likewise, the pCVD at the ODH-unaffected hemiretina, cpVD at the ODH-unaffected hemiretina, and cpRNFLT at the ODH-unaffected quadrant in the ODH+ eye was also similar to those in ODH− eye, i.e., 42.14% vs. 42.28%, 50.7% vs. 51.6%, and 130 μm vs. 138 μm, respectively.

Figure 4 presents the findings for a representative pair of matched OAG eyes with and without ODH in our current study series, in which the ODH+ eye showed a lower pCVD at ODH-affected hemiretina, lower cpVD at the ODH-affected sector, and thinner cpRNFLT at the ODH affected clock hour compared with the ODH− eye.

## 4. Discussion

We have here evaluated the impact of ODH on pCVD loss within the β-PPA zone in early-stage OAG eyes. On OCTA scans, the pCVD measurements were significantly lower in the ODH+ eyes than in the ODH− eyes at ODH-affected hemiretinae (*p* < 0.05), whereas there were no significant differences in global pCVD and pCVD values at ODH-unaffected hemiretinae between the ODH+ and ODH− eyes (both *p* > 0.05). Moreover, the presence of ODH was significantly associated with lower pCVD at ODH-dominant inferior hemiretinae based on our multivariate analyses. From the results of logistic regression analyses, a reduced pCVD at ODH-affected hemiretinae was significantly associated with the presence of ODH. These findings suggest that ODH+ eyes may show microvasculature loss confined to the hemiretinal region where ODH is located in the parapapillary choroidal layer. To the best of our knowledge, our present study is the first to evaluate the geographic pattern of pCVD loss (i.e., regional VS. global) within the β-PPA zone in early–stage OAG eyes with ODH.

The regional cpRNFLT was significantly lower in the ODH+ group in our present series than in the ODH− group at the ODH-affected clock-hour sector, while there were no significant differences in the global cpRNFLT or cpRNFLT at the ODH-unaffected clock-hour sector between the two groups, despite the similar age and glaucoma severity in our patients. These findings are in line with the results of a previous study which reported that ODHs are spatially associated with localized structural changes such as RNFL defects [30]. Suh et al. [31] also previously reported localized thinning of RNFL at the location of the ODH. With regard to cpVD measurements, we found that ODH+ eyes showed significantly lower regional cpVDs (at the 45° sector and hemiretinae) than ODH− eyes at the ODH-affected locations. Similar to the cpRNFLT variable, there were no significant differences in cpVD measurements between the ODH+ and ODH− groups at the global and ODH-unaffected sectors. Our current findings are therefore consistent with earlier results in which a reduction in the cpVD at the peripapillary sector with ODH was shown to be significantly greater than that of peripapillary sector without ODH [32]. Of interest, when the pCVD was measured regionally at each hemiretina as well as globally in the choroidal layer using OCTA in our present cohort, we found that the pCVD values were significantly lower in the ODH+ group at ODH-affected hemiretinae, despite no significant differences in the global pCVD and pCVD at ODH-unaffected hemiretinae between the ODH+ and ODH− groups (both *p* > 0.05, Table 2). These observations suggest that glaucomatous eyes with ODH demonstrate more severe regional microvasculature loss adjacent to or at the location of the ODH.

We found by multivariate analysis that the presence of CMvD had negative relationship with global pCVD value in the entire group of OAG eyes (Table 3). Conversely, global cpVD had positive relationship with global pCVD value. This finding may imply that the lower global pCVD value, which may be indicative of a poor microvascular perfusion around the ONH, can lead to greater degree of glaucomatous damage [33], resulting in a significant positive correlation between the global cpVD and pCVD, as shown in Table 3.

Regionally, the presence of CMvD showed significant associations with pCVD in both the inferior and superior hemiretinae (Table 4). These findings are in agreement with previous study by Jo et al. [10] reporting that the glaucomatous eyes with CMvD showed globally reduced pCVD values within the β-PPA due to the presence of an extensive interconnection of microvasculature networks of parapapillary choroid. In contrast, OAG eyes with ODH did not show a similar global reduction in pCVD to that which was noted with CMvD in the current study. In fact, the presence of ODH was significantly associated with a reduction in pCVD only at the inferior hemiretinae where ODH was predominantly located in ODH+ eyes (*n* = 37, 74%). It is noteworthy, however, that there were no associations between the detection of ODH and pCVD loss at the superior hemiretinae (ODH-deficient hemiretinae) as well as global zone (Table 3 and Table 4). One possible explanation for our findings is that the pathogenic mechanism of ODH may be different from that of CMvD, even though both conditions often coexist in glaucomatous eyes. The occurrence of ODH may be related to the localized vascular event at the level of LC or RNFL in the vicinity of or at the location of ODH [16,17]. In support of this speculation, ODH shows topographic correlation with glaucomatous structural damage such as β-PPA, increased CDR, LC defects, and progressive RNFL thinning in the location of ODH [14,16,17]. Another explanation is that the regional reduction in pCVD may be secondary to localized progressive axonal damage and loss of neural tissue following ODH and subsequent decline in the metabolic needs at the corresponding or in the vicinity of ODH locations.

Previous studies have revealed that eyes with longer AL showed thinner choroidal thickness as well as lower choroidal VD [34,35,36]. Alshareef et al. [35] reported that the choroidal VD has a strong negative relationship with AL. These previous findings are consistent with our current results, which show significant associations between a longer AL and lower pCVD noted at the inferior hemiretinae as well as global area (Table 4).

Logistic regression analysis indicated that a lower pCVD at ODH-affected hemiretinae, thinner cpRNFLT at an ODH-affected clock hour, and lower cpVD at ODH-affected sectors were significantly associated with the presence of ODH (*p* < 0.05, Table 5), while the global pCVD, cpRNFLT and cpVD were not related to ODH. These results suggest that ODH occurs at the region of structural and microvasculature damage in the ONH/RNFL. Although the temporal relationship is unclear based on our present study design, one plausible hypothesis is that localized blood flow changes may occur at the location of ONH/RNFL damage and result in subsequent development of localized ODH and progressive glaucomatous damage. Park et al. [37] reported previously in this regard that ODH occurring at the margins of RNFL defects had accompanying hemodynamic changes, which prolonged the arteriovenous transit time and vessel filling defect. These authors suggested that the course of the microvessels may be curved or kinked, resulting in blood flow stasis and the formation of thrombosis that may contribute to the occurrence of ODH.

An ODH is usually located in inferior temporal or superior temporal region of the optic disc [38,39,40]. Likewise, CMvDs are most often observed at the inferior temporal region of the β-PPA zone [41,42]. Our present study results also showed that ODHs were most frequently located at the inferior temporal optic disc region followed by the superior temporal region (33 eyes (66%) vs. nine eyes (18%), respectively; Figure 3A). Of interest from this perspective, CMvDs were mostly observed at the inferior temporal region, (17 eyes, 89.5%), but there was no CMvD detected at superior temporal region in our current study series (Figure 3A). These findings again suggest that the pathogenic mechanism of ODH may differ from that of CMvD. In other words, CMvD may occur at the location of the greatest severity of glaucomatous damage, whereas ODH can arise anywhere that structural and/or vascular damage occurs in the ONH. Notably, however, two thirds of the CMvDs in our current cohorts showed spatial concordance with ODHs (63.2%; Figure 3B). The association between ODH and CMvD could be explained by the vascular mechanism of glaucomatous optic nerve injury. Short posterior ciliary (SPC) arteries supply both the choroid within the parapapillary area and ONH structures, including the prelaminar tissue and LC [7,8]. Hence, a microvasculature insufficiency in the choroid and ONH may lead to the formation of both ODH and CMvD by influencing not only retinal/ONH perfusion, leading to ODH, but also microvasculature changes in the choroidal layer, which may lead to the development of CMvD [2]. Rao et al. [21] hypothesized previously that ODH and CMvD could be the result of vascular phenomena such as small vessel disease or the obliteration of choriocapillaris lobules.

Our current study had some limitations of note. First, the OAG patients we assigned to the ODH− group in the analyses could have had ODH in the past when they were not monitored or in the period between study visits. Second, the en face choroidal images of OCTA may have had substantial artifacts inherent to current technology. It is sometimes difficult to obtain uniform focus across the surface of the optic disc and choroidal layer around the ONH. Additionally, projection artifacts, such as signals from the superficial retinal vessels and ODH projecting onto the choroidal slab, may influence pCVD and CMvD assessments. Nonetheless, eyes with poor-quality images and artifacts were excluded from our cohorts and large retinal vessels and/or ODH within the β-PPA were excluded on scanning laser ophthalmoscopy images during the assessment of pCVD and CMvD. Third, as our study evaluated early-stage OAG eyes with VF defects confined to a single hemifield and ODH isolated to one hemiretina, our data may be subject to selection bias and limit the generalizability of our findings. However, early-stage OAG eyes with ODH were selected in an effort to best evaluate the relationship between ODH and regional vs. global pCVD loss, while avoiding the confounders such as disease severity. Fourth, we recruited only Korean patients who visited our university hospital. As patients in a tertiary university practice may have different characteristics than those observed in the general population, our results should be cautiously interpreted in terms of their overall clinical relevance, including other ethnic populations. Fifth, the ODH-affected and –unaffected regions for the regional relationship between ODH and OCT/OCTA parameters used in our analyses included pCVD hemiretinae, cpRNFLT quadrants and clock hours, and cpVD 45° sector, which were not the same topographically. Nonetheless, these regions are those that are topographically closest to each other, most frequently affected by early-stage glaucoma, and automatically reported by the OCT/OCTA devices used in our study. Moreover, there is currently no automatic report for the regional pCVD readings provided by OCTA device. Sixth, our cross-sectional study design did not provide information on the temporal relationship between ODH and pCVD/cpRNFLT/cpVD changes in the ODH+ eyes in our study. This association needs to be investigated in a future prospective longitudinal study. Seventh, 90% of our patients (*n* = 90) had normal-tension glaucoma (NTG), while 10% had primary open-angle glaucoma (POAG, *n* = 10). Therefore, our findings may not apply to those eyes with different types of glaucoma (i.e., POAG or angle-closure glaucoma). Eighth, since ODH was confirmed based on the presence of ODH at the time of OCTA evaluation or anytime in the past by reviewing the past medical records, the time of ODH occurrence and OCTA measurement may not have been concurrent in some eyes of our study. However, the average follow-up period for the ODH+ eyes was only 0.51 year, which indicates that there was a direct relationship between ODH occurrence and OCTA values that were analyzed. Lastly, the normal healthy group consisted of patients from the general eye clinic who had undergone comprehensive ocular examinations including VF test as they had a family history of glaucoma or subjective VF abnormality symptoms. Therefore, the normal healthy subjects in our study may not comprise of true normal healthy volunteers.

In conclusion, the pCVD values were significantly lower in the ODH+ eyes than in the ODH− eyes at ODH-affected hemiretinae, whereas there were no significant differences in global pCVD and pCVD values at ODH-unaffected hemiretinae between the two groups. ODH was significantly associated with a greater degree of pCVD reduction at ODH-affected hemiretinal region in early-stage OAG eyes, indicating that ODH may be related to regional microvasculature loss in the parapapillary choroid.

## Figures and Tables

**Table 1:** Baseline Characteristics of Patients with Open-Angle Glaucoma Eyes, with and without Optic Disc Hemorrhage, and Healthy Eyes.

Characteristic	A. OAG Eyes with ODH (*n* = 50)	B. OAG Eyes without ODH (*n* = 50)	C. Healthy Eyes (*n* = 50)	*p* Values (Post Hoc A vs. B, A vs. C, B vs. C)
Age, year	59.40 ± 13.42	59.02 ± 9.62	56.48 ± 10.29	0.371
Male: female ratio	18:32	21:19	17:33	0.593 *
IOP, mmHg	14.24 ± 2.72	15.18 ± 3.11	14.46 ± 2.98	0.251
CCT, μm	535.61 ± 39.93	534.30 ± 27.49	541.23 ± 40.84	0.614
SE, diopter	−1.43 ± 2.30	−1.19 ± 2.17	−0.89 ± 1.79	0.499
AL, mm	24.36 ± 1.15	24.05 ± 1.08	24.22 ± 0.98	0.416
Hypertension, *n* (%)	9 (18%)	8 (16%)	10 (20%)	0.873 *
Diabetic mellitus, *n* (%)	5 (10%)	8 (16%)	3 (6%)	0.265 *
SBP, mmHg	123.03 ± 10.40	121.00 ± 18.16	122.87 ± 9.51	0.778
DBP, mmHg	72.73 ± 9.28	74.72 ± 9.28	72.01 ± 8.02	0.308
MOPP, mmHg	45.32 ± 5.64	45.10 ± 7.59	44.58 ± 4.72	0.885
Eye drops, *n*	1.02 ± 0.73	1.04 ± 1.08	0.00 ± 0.00	**<0.001** † (0.991, **<0.001**, **<0.001**)
Visual field MD, dB	−3.08 ± 1.59	−3.05 ± 1.51	0.10 ± 1.30	**<0.001** † (0.955, <0.001, **<0.001**)
Affected VF hemifield, S:I	31:19	31:19	-	1.000 *
ODH sites, S:I	13:37	-	-	
CMvD, *n* (%)	19 (38%)	9 (18%)	0 (0%)	**<0.001** ‡ **(<0.001**, 0.881, **<0.001**)
Follow-up period for the review of medical history, year	0.51 ± 1.21	4.19 ± 4.36		

AL, axial length; CCT, central corneal thickness; CMvD, choroidal microvasculature dropout; dB, decibel; DBP, diastolic blood pressure; I, inferior; IOP, intraocular pressure; MD, mean deviation; MOPP, mean ocular perfusion pressure; *n*, number; ODH, optic disc hemorrhage; S, superior; SBP, systolic blood pressure; SE, spherical equivalent; VF, visual field. Data are reported as a mean ± standard deviation, number (%), or ratio. Statistically significant differences (*p* < 0.05) are indicated in bold type. † One-way ANOVA tests with Tukey’s correction as post hoc analysis. * Chi-square test. ‡ Chi-square test with Bonferroni correction as post hoc analysis.

**Table 2:** Comparisons of OCT and OCTA Parameters in 100 Eyes with Open-Angle Glaucoma.

	A. OAG Eyes with ODH (*n* = 50)	B. OAG Eyes without ODH (*n* = 50)	C. Healthy Eyes (*n* = 50)	*p* Values (Post Hoc A vs. B, A vs. C, B vs. C)
Global cpRNFLT	80.14 ± 8.73	82.94 ± 8.78	100.38 ± 8.48	**<0.001** (0.242, **<0.001**, **<0.001**)
cpRNLFT at ODH-affected clock-hour	65.86 ± 12.95	76.48 ± 20.15	111.34 ± 26.97	**<0.001** (**0.030**, **<0.001**, **<0.001**)
cpRNFLT at ODH-unaffected clock-hour	89.62 ± 26.71	90.23 ± 26.81	114.02 ± 24.18	**<0.001** (0.993, **<0.001**, **<0.001**)
cpRNFLT at ODH-affected quadrant	84.30 ± 14.80	86.90 ± 15.59	100.88 ± 9.70	**<0.001** (0.607, **<0.001**, **<0.001**)
cpRNFLT at ODH-unaffected quadrant	91.94 ± 21.58	91.54 ± 18.05	101.00 ± 9.26	**0.009** (0.992, **0.024**, **0.018**)
Global cpVD	46.06 ± 4.24	46.30 ± 3.80	51.85 ± 2.66	**<0.001** (0.943, **<0.001**, **<0.001**)
cpVD at ODH-affected sector	37.67 ± 10.39	43.79 ± 11.32	54.53 ± 4.36	**<0.001** (**0.004**, **<0.001**, **<0.001**)
cpVD at ODH-unaffected sector	46.32 ± 10.52	46.47 ± 8.41	54.74 ± 2.95	**<0.001** (0.995, **<0.001**, **<0.001**)
cpVD at ODH-affected hemiretinae	44.11 ± 5.77	46.31 ± 4.47	52.29 ± 2.39	**<0.001** (**0.039**, **<0.001**, **<0.001**)
cpVD at ODH-unaffected hemiretinae	47.35 ± 4.49	47.67 ± 3.84	51.40 ± 3.67	**<0.001** (0.916, **<0.001**, **<0.001**)
Global pCVD	41.14 ± 1.63	41.78 ± 2.26	44.30 ± 2.03	**<0.001** (0.240, **<0.001**, **<0.001**)
pCVD at ODH-affected hemiretinae	39.96 ± 2.30	41.70 ± 2.30	44.29 ± 2.10	**<0.001** (**0.001**, **<0.001**, **<0.001**)
pCVD at ODH-unaffected hemiretinae	42.05 ± 1.50	42.31 ± 2.34	44.33 ± 2.10	**<0.001** (0.799, **<0.001**, **<0.001**)

cpRNFLT, circumpapillary retinal nerve fiber layer thickness; cpVD, circumpapillary vessel density; ODH, optic disc hemorrhage; pCVD, parapapillary choroidal vascular density. Data are reported as a mean ± standard deviation. Results were compared by one-way ANOVA tests with Turkey correction. Statistically significant differences (*p* < 0.05) are indicated in bold type.

**Table 3:** Univariate and Multivariate Linear Regression Analyses of Factors Associated with Global Parapapillary Choroidal Vascular Density in 100 Eyes with Open-Angle Glaucoma.

	Univariate Analysis			Multivariate Model 1 With Global cpVD, CMvD, AL			Multivariate Model 2 With Global RNFLT, CMvD, AL		
	β-Coefficient	95% CI	*p* Value	β-Coefficient	95% CI	*p* Value	β-Coefficient	95% CI	*p* Value
Age	0.001	−0.033 to 0.035	0.953						
IOP	0.084	−0.051 to 0.218	0.219						
CCT	−0.003	−0.015 to 0.009	0.610						
ODH presence	−0.647	−1.431 to 0.137	0.105						
CMvD presence	−2.048	−2.832 to −1.264	**<0.001**	−1.940	−2.820 to −1.060	**<0.001**	−2.082	−2.943 to −1.220	**<0.001**
AL	−0.362	−0.653 to −0.075	**0.014**				−0.260	−0.514 to −0.007	**0.044**
SBP	0.019	−0.011 to 0.050	0.210						
DBP	0.034	−0.017 to 0.086	0.191						
MOPP	0.035	−0.036 to 0.103	0.337						
VF MD	0.071	−0.187 to 0.328	0.587						
Global cpRNFLT	0.051	0.007 to 0.095	**0.023**						

AL, axial length; CCT, central corneal thickness; CI, confidence interval; cpRNFLT, circumpapillary retinal nerve fiber layer thickness; cpVD, circumpapillary vessel density; CMvD, choroidal microvasculature dropout; DBP, diastolic blood pressure; I, inferior; IOP, intraocular pressure; MD, mean deviation; MOPP, mean ocular perfusion pressure; ODH, optic disc hemorrhage; S, superior; SBP, systolic blood pressure; SE, spherical equivalent; VF, visual field. Statistically significant differences (*p* < 0.05) are indicated in bold type.

**Table 4:** Univariate and Multivariate Linear Regression Analyses of Factors Associated with pCVD at Inferior and Superior Hemiretinae in 100 Open-Angle Glaucoma Eyes.

	**Univariate Analysis**			**Multivariate Model 1** **With Global cpVD, ODH, CMvD, AL**			**Multivariate Model 2** **With Global cpRNFLT, ODH, CMvD, AL**		
	**β-Coefficient**	**95% CI**	***p***	**β-Coefficient**	**95% CI**	***p***	**β-Coefficient**	**95% CI**	***p***
**Inferior hemiretinae**									
Age	0.007	−0.033 to 0.048	0.718						
IOP	0.113	−0.044 to 0.271	0.156						
CCT	−0.004	−0.019 to 0.010	0537						
ODH presence	−1.689	−2.559 to −0.820	**<0.001**	−1.085	−1.912 to −0.258	**0.011**	−1.085	−1.912 to −0.258	**0.011**
CMvD presence	−2.696	−3.584 to −1.809	**<0.001**	−2.517	−3.476 to −1.559	**<0.001**	−2.517	−3.476 to −1.559	**<0.001**
AL	−0.502	−0.840 to −0.164	**0.004**	−0.324	−0.606 to 0.043	**0.025**	−0.342	−0.606 to −0.043	**0.025**
SBP	0.015	−0.021 to 0.052	0.404						
DBP	0.019	−0.042 to 0.081	0.535						
MOPP	0.008	−0.075 to 0.090	0.852						
VF MD	0.084	−0.218 to 0.387	0.581						
Global cpRNFLT	0.063	0.011 to 0.114	**0.018**						
Global cpVD	0.146	0.032 to 0.259	**0.012**						
	**Univariate Analysis**			**Multivariate Model 1** **With Global cpVD, CMvD, AL**			**Multivariate Model 2** **With Global cpRNFLT, CMvD, AL**		
	**β** **-Coefficient**	**95% CI**	***p***		**β** **-Coefficient**	**95% CI**	***p***		**β** **-Coefficient**
**Superior hemiretinae**									
Age	0.007	−0.029 to 0.043	0.710						
IOP	0.068	−0.075 to 0.210	0.048						
CCT	−0.004	−0.016 to 0.009	0.557						
ODH presence	−0.105	−0.940 to 0.731	0.804						
CMvD presence	−1.828	−2.679 to −0.976	**<0.001**	−1.526	−2.528 to −0.525	**0.003**	−1.975	−2.972 to −0.979	**<0.001**
AL	−0.353	−0.673 to −0.033	**0.031**						
SBP	0.016	−0.017 to 0.049	0.330						
DBP	0.027	−0.028 to 0.082	0.338						
MOPP	0.024	−0.050 to 0.098	0.515						
VF MD	−0.010	−0.281 to 0.260	0.939						
Global cpRNFLT	0.054	0.002 to 0.095	**0.040**						
Global cpVD	0.210	0.112 to 0.307	**<0.001**	0.167	0.052 to 0.282	**0.005**			

AL, axial length; CCT, central corneal thickness; CI, confidence interval; cpRNFLT, circumpapillary retinal nerve fiber layer thickness; cpVD, circumpapillary vessel density; CMvD, choroidal microvasculature dropout; DBP, diastolic blood pressure; I, inferior; IOP, intraocular pressure; MD, mean deviation; MOPP, mean ocular perfusion pressure; ODH, optic disc hemorrhage; S, superior; SBP, systolic blood pressure; SE, spherical equivalent; VF, visual field. Statistically significant differences (*p* < 0.05) are indicated in bold type.

**Table 5:** Univariate and Multivariate Logistic Regression Analysis of Factors Associated with Optic Disc Hemorrhage in 100 Eyes with Open-Angle Glaucoma.

	Univariate Analysis			Multivariate Model 1 With pCVD-Affected Hemiretinae and CMvD			Multivariate Model 2 With cpVD-Affected Sector, cpVD-Affected Hemiretinae and CMvD			Multivariate Model 3 With cpRNFLT-Affected Clock-Hour and CMvD		
	OR	95% CI	*p*	OR	95% CI	*p*	OR	95% CI	*p*	OR	95% CI	*p*
Age	1.003	0.969 to 1.038	0.862									
IOP	0.906	0.788 to 1.041	0.165									
CMvD presence	2.792	1.113 to 7.007	**0.029**									
AL	1.105	0.836 to 1.462	0.483									
SBP	1.007	0.976 to 1.040	0.648									
DBP	0.965	0.914 to 1.018	0.189									
MOPP	0,999	0.932 to 1.070	0.972									
VF MD	0.992	0.783 to 1.258	0.950									
Global pCVD	0.845	0.689 to 1.037	0.107									
pCVD at affected hemiretinae	0.713	0.586 to 0.868	**0.001**	0.713	0.586 to 0.868	**0.001**						
pCVD at unaffected hemiretinae	0.935	0.764 to 1.144	0.512									
Global cpRNFLT	0.964	0.920 to 1.009	0.115									
cpRNFLT at affected clock hour	0.962	0.937 to 0.988	**0.004**							0.965	0.939 to 0.991	**0.009**
cpRNFLT at unaffected clock hour	0.999	0.984 to 1.014	0.911									
cpRNFLT at affected quadrant	0.982	0.950 to 1.015	0.287									
cpRNFLT at unaffected quadrant	0.984	0.951 to 1.019	0.373									
Global cpVD	0.985	0.893 to 1.087	0.767									
cpVD at affected sector	0.949	0.913 to 0.987	**0.008**				0.955	0.918 to 0.994	**0.024**			
cpVD at unaffected sector	0.998	0.956 to 1.042	0.937									
cpVD at affected hemiretinae	0.914	0.837 to 0.998	**0.044**									
cpVD at unaffected hemiretinae	0.982	0.891 to 1.081	0.705									

AL, axial length; CCT, central corneal thickness; CI, confidence interval; cpRNFLT, circumpapillary retinal nerve fiber layer thickness; cpVD, circumpapillary vessel density; CMvD, choroidal microvasculature dropout; DBP, diastolic blood pressure; I, inferior; IOP, intraocular pressure; MD, mean deviation; MOPP, mean ocular perfusion pressure; S, superior; SBP, systolic blood pressure; SE, spherical equivalent; VF, visual field. Statistically significant differences (*p* < 0.05) are indicated in bold type.

## Data Availability

Data collected for this study, including individual patient data, will not be made available.
